# Congenital ciliary body cysts causing lens abnormalities and secondary angle closure glaucoma in a child

**DOI:** 10.1016/j.ajoc.2022.101723

**Published:** 2022-10-14

**Authors:** Marcus L. Turner, Alejandra G. de Alba Campomanes, Jay M. Stewart, Julius T. Oatts

**Affiliations:** Department of Ophthalmology, University of California, San Francisco, San Francisco, CA, USA

**Keywords:** Ciliary body cyst, Childhood glaucoma, Secondary childhood glaucoma, Microspherophakia, Ectopia lentis, Ultrasound biomicroscopy

## Abstract

**Purpose:**

To report a case of congenital ciliary body cysts causing microspherophakia, ectopia lentis, and secondary angle closure glaucoma in an infant.

**Observations:**

A 16-month-old male was found to have bilateral ciliary body cysts associated with zonular laxity or absence causing microspherophakia and ectopia lentis as demonstrated on multimodal imaging. Additionally, the patient had secondary angle closure glaucoma which was likely multi-factorial related to both lens abnormalities and anterior displacement of the iris from the cysts themselves. The patient underwent lensectomy and cyst removal which resulted in intraocular pressure stabilization and visual rehabilitation.

**Conclusions and Importance:**

Congenital ciliary body cysts are a rare cause of lens abnormalities and secondary angle closure glaucoma in children. Information regarding genetic underpinnings or systemic associations is limited.

## Highlights

• Ciliary body cysts can be a cause of secondary angle closure glaucoma in children.
• Multimodal imaging is a useful aid in the diagnosis of secondary glaucoma in children.
• Ciliary body cysts can lead to zonular and lens changes causing glaucoma.
• Surgery may be required in ciliary body cysts causing glaucoma.

## 1 Introduction

Childhood glaucoma is typically categorized by etiology and can be primary or secondary.^1^ Primary congenital glaucoma is due to developmental abnormalities of the anterior chamber angle, while secondary glaucoma occurs in the setting of other co-existing ocular or systemic disease. Secondary glaucoma is the most common cause of glaucoma in children, accounting for as much as 45% of cases.^2^ The most common causes of secondary glaucoma are glaucoma following cataract surgery and glaucoma following trauma.^2^ Secondary angle closure glaucoma in children is rare, with only a small number of cases previously reported.^3^, ^4^, ^5^, ^6^, ^7^ While approximately 90% of angle closure glaucoma in adults is due to pupillary block, in children, angle closure is primarily due to structural or developmental ocular abnormalities.^3^^,^^8^ A small study of angle closure in young patients reported diagnoses in order of frequency as: plateau iris syndrome, iridociliary cysts, retinopathy of prematurity, uveitis, nanophthalmos, pupillary block, Weill-Marchesani syndrome, Marfan syndrome, miotic-induced angle closure, persistent hyperplastic primary vitreous, and idiopathic lens subluxation.^3^

Ciliary body cysts are typically unilateral, discovered incidentally, and rarely cause complications.^9^ We report a case of a child with bilateral secondary angle closure glaucoma in the setting of lens abnormalities and ciliary body cysts. We demonstrate how multimodal imaging can help determine the cause of secondary angle closure glaucoma in children.

## 2 Case report

A 16-month-old male with a history of hypotonia, gross motor and speech delay, obstructive sleep apnea, and somatic overgrowth (>97th percentile for height and weight) was referred to the pediatric ophthalmology service due to parental concerns for delayed eye contact and abnormal eye movement since birth. He had no history of tearing, photophobia, or blepharospasm. He was an ex-full term infant born via emergency C-section due to reduced fetal heart rate. His maternal great grandfather had adult-onset glaucoma and family ocular history was otherwise unremarkable.

On examination, the vision was central, steady, and maintained in the right eye and uncentral, unsteady and unmaintained in the left eye. There was a moderate angle intermittent exotropia with latent nystagmus in the left eye. Intraocular pressure (IOP) was 32 mmHg in the right eye and 33 mmHg in the left eye by iCare tonometry. Portable slit lamp examination was notable for white and quiet conjunctiva, clear corneas with enlarged horizontal diameters, and shallowing of both anterior chambers with peripheral anterior bowing of the iris. There appeared to be microspherophakia in both eyes and nuclear lens opacities in the left worse than right eyes. Dilated fundus examination revealed significant optic nerve cupping in both eyes (vertical cup:disc ratio 0.7 right eye, 0.9 left eye) and was otherwise grossly normal, though limited by patient cooperation. Cycloplegic refraction was −13.50 + 3.50 × 020 in the right eye and −17.50 + 2.00 × 075 in the left eye. Due to the elevated IOP and difficult examination, the patient was started on latanoprost and dorzolamide-timolol eye drops in both eyes and referred for an examination under anesthesia.

**Fig. 1:**
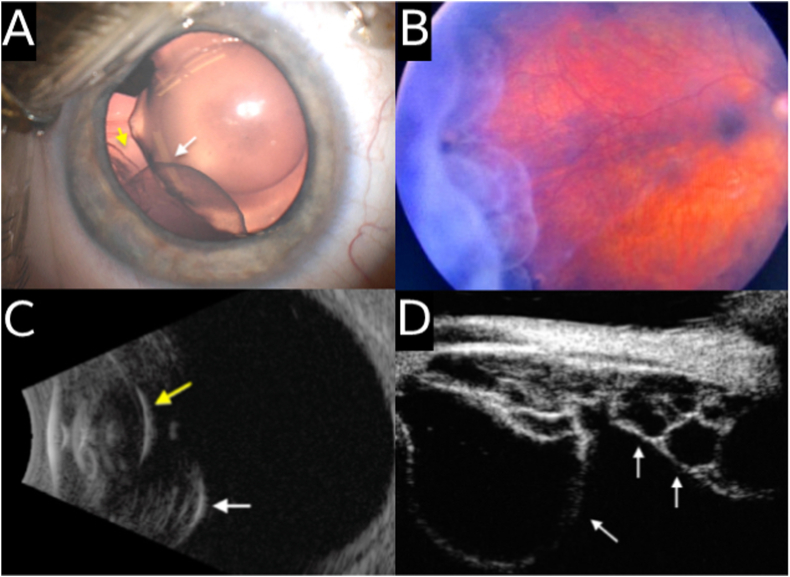
Multimodal imaging of the right eye of an infant with multiple ciliary body cysts. (A) Surgical microscopic photograph demonstrating ectopia lentis (white arrow) secondary to abnormal zonules presumably primarily caused by extensive temporal ciliary body cysts (yellow arrow). (B) Dilated fundus photography demonstrating multiple clear, thin-walled ciliary body cysts extending into the vitreous cavity. (C) B-scan ultrasound demonstrating multiple thin-walled cystic structures originating from the ciliary body (white arrow) posterior to and abutting the posterior lens capsule (yellow arrow). (D) Ultrasound biomicroscopic imaging of the right eye showing multiple ciliary body cysts of varying sizes (white arrows). (For interpretation of the references to colour in this figure legend, the reader is referred to the Web version of this article.)

**Fig. 2:**
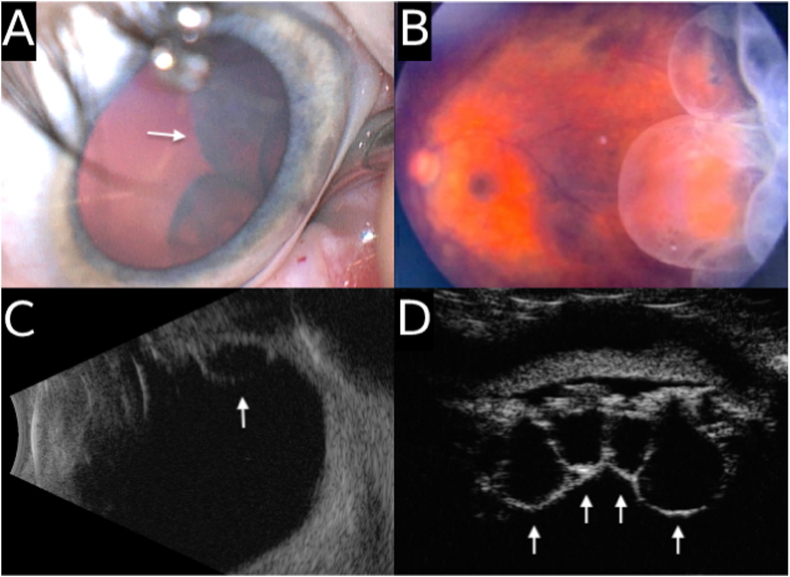
Multimodal imaging of the left eye of an infant with multiple ciliary body cysts. (A) Surgical microscopic photograph with scleral depression demonstrating extensive temporal ciliary body cysts (white arrow). (B) Dilated fundus photography demonstrating multiple large, clear, thin-walled ciliary body cysts extending into the vitreous cavity. (C) B-scan ultrasound demonstrating thin-walled cystic structures originating from the ciliary body (white arrow). (D) Ultrasound biomicroscopic imaging of the left eye showing multiple ciliary body cysts of varying sizes (white arrows).

Under anesthesia, the IOP was 27.5 mmHg in the right eye and 23.5 mmHg in the left eye and the above examination findings were confirmed. Gonioscopy showed no visible structures in the right eye, though the angle opened up to the ciliary body band 360° with compression gonioscopy. The left eye angle was open to the ciliary body band except for an area of temporal peripheral anterior synechiae (PAS) from 3 to 4 o'clock. Additionally, on detailed dilated examination, he was noted to have numerous bilateral temporal ciliary body cysts extending into the vitreous cavity. These cysts appeared to be pushing the iris forward causing angle closure, disrupting the lens and lens zonule anatomy, and contributing to the abnormal lens morphology (Fig. 1A and B). B-scan ultrasonography of both eyes with a 10 MHz probe demonstrated multiple thin-walled cystic structures originating from the ciliary body behind and adjacent to the posterior lens capsule (Figs. 1C and 2C). Ultrasound biomicroscopy (UBM) with a 40 MHz probe confirmed these findings and showed large temporal ciliary body cysts extending into the vitreous cavity and microspherophakia with central lens opacities in both eyes (Figs. 1D and 2D). The overall assessment was secondary angle closure glaucoma with ectopia lentis and microspherophakia in both eyes in the setting of ciliary body cysts disrupting lens, zonule, and anterior chamber anatomy. The decision was made to continue medical treatment and return to the operating room with a retina specialist to evaluate the cysts and consider the role of simultaneous cyst and lens removal.

**Fig. 3:**
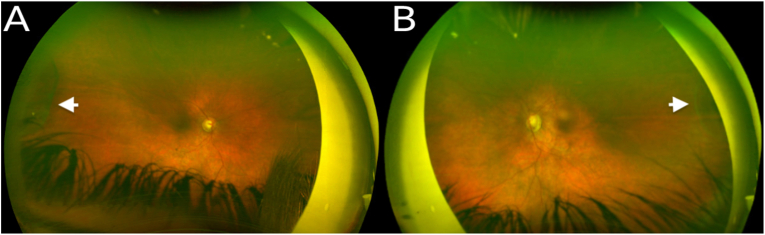
Wide-field dilated fundus photography demonstrating flat, far-peripheral temporal ciliary body cyst remnants with minimal protrusion into the vitreous cavity (white arrow) and subtle non-specific retinal pigmentary changes in the macula in both eyes. Stable glaucomatous optic nerve cupping in the left (B) > right (A) eye.

The next month, with a retina surgeon, lensectomy, capsulectomy, vitrectomy, and cyst excision were performed in the right eye. The cyst was removed using the vitrector as well as intraocular scissors and forceps. A portion of the cyst wall was sent for pathology, but given the tissue friability, it did not survive processing. The following month, the patient underwent the same procedure in the left eye. Post-operatively, he was managed with glasses and patching for refractive and strabismic amblyopia. His IOP in the left eye remained elevated with progression of optic nerve cupping on maximum topical medications so he underwent vitrectomy with endoscopic cyclophotocoagulation in the left eye at 3 years of age. The glaucomatous progression in the left eye was likely secondary to the extensive peripheral anterior synechiae which formed prior to cyst removal. At most recent follow-up, one year after cyst excision, the patient was 4 years of age. His best corrected visual acuity was 20/60 in the right eye and 20/250 in the left eye by LEA symbols. IOP was 16 mmHg in the right eye and 17 mmHg in the left eye on dorzolamide-timolol twice daily in both eyes and latanoprost in the evening in the right eye. His anterior chamber remained deep with some temporal PAS in the left worse than right eyes. His cycloplegic refraction was stable at +12.25 + 1.00 × 138 in the right eye and +12.50 + 0.50 × 115 in the left eye. He demonstrated stable, flat, far-peripheral temporal ciliary body cyst remnants bilaterally with minimal protrusion into the vitreous cavity, stable glaucomatous optic nerve cupping in the left > right eye, and non-specific retinal pigmentary changes in the macula of both eyes (Fig. 3).

The patient was evaluated by the genetics team who performed a congenital cataract panel (39 genes, Invitae) and an ectopia lentis panel (14 genes, Blueprint) which were both negative. Whole exome sequencing with mitochondrial genome (XomeDxplus, trio) was negative with no pathogenic variants to explain the clinical phenotype and no secondary findings.

## 3 Discussion

We report a case of ciliary body cysts as an unusual cause of secondary angle closure glaucoma and lens abnormalities in an infant with no identifiable underlying genetic change. Secondary glaucoma in children has been reported in the setting of congenital ocular abnormalities including anterior segment dysgenesis, metabolic disorders, phakomatoses, mitotic disorders, as well as acquired conditions such as tumors, uveitis, trauma, and steroid use.^10^^,^^11^ Secondary glaucoma in children is usually open-angle. A narrow or closed angle is uncommon and warrants further investigation, usually with an examination under anesthesia and B-Scan ultrasonography and/or UBM as helpful testing modalities.^12^^,^^13^

The true prevalence of ciliary body cysts is unknown, though UBM studies of incidental iridociliary lesions in asymptomatic adults have reported a prevalence between 15 and 54%, which may decrease with age.^13^^,^^14^ Of iridociliary cysts, one report found that the majority of cysts originate from the iris pigment epithelium (84.4%), with the remainder originating from the ciliary body (3.8%), and 0.9% involved both the ciliary body and the pars plana, demonstrating the greater rarity of ciliary body cysts.^15^ Ciliary body cysts can be primary, occurring spontaneously and consisting of epithelial-lined spaces arising from the epithelial layer; or secondary, arising from traumatic implantation of the epithelium, long term use of miotics, parasitic lesions, or metastatic disease.^16^ Multimodal imaging, specifically UBM, can help differentiate the causes of iridociliary cysts, with classic findings of ciliary body cysts including well-defined, thin walls and clear intracavitary fluid.^13^^,^^17^^,^^18^

While ciliary body cysts are typically clinically insignificant, prior reports have noted that large or multiple ciliary body cysts can displace the peripheral iris anteriorly leading to angle closure glaucoma.^16^^,^^19^ Multiple studies have described angle closure in the setting of disrupted lens zonule anatomy, leading to abnormal lens configuration or position.^20^, ^21^, ^22^, ^23^ Given the examination and imaging findings in this patient, the ciliary body cysts, in combination with the lens abnormalities, were the essential contributor to the angle closure.

Prior reports of similar ciliary body cyst findings are sparse. A study from 1984 described four cases of angle closure with PAS due to ciliary body cysts.^4^ Unlike our patient, these cases ranged in age from 36 to 55 years, occurred in an autosomal dominant inheritance pattern, and all four patients were female. A report from 1996 was the first to note plateau iris syndrome occurring in three adult patients caused by ciliary body cysts.^24^ Notably, our pediatric case featured neither plateau nor pseudoplateau iris. Another case describing a congenital retrolenticular cyst in a toddler was the first report of a cyst in such a young child, however the cyst was found to have originated from the vitreous.^25^ An additional case report of a young child with a large unilateral ciliary body cyst causing lens subluxation and damage to zonule fibers also reported co-occurrence of multiple congenital iris cysts.^26^ While this case was similar to ours, the findings in their patient were unilateral and not associated with any glaucomatous optic nerve changes. Finally, a recent report described a case of extrascleral extension of a ciliary body cyst in a 3-year-old girl, describing a rare complication of this condition.^27^

Lastly, there are several genetic syndromes that demonstrate ocular phenotypic overlap with our patient. Marfan syndrome, an autosomal dominant disorder, is commonly associated with eye manifestations including myopia, ectopia lentis, and glaucoma, although it is also accompanied by skeletal abnormalities and is detectable on genetic testing.^11^ Weill-Marchesani syndrome, a rare genetic connective tissue disorder, can present with hypotonia, microspherophakia, partial presence of zonules, ectopia lentis, cataracts, shallow anterior chambers, and glaucoma; yet genetic testing for the ADAMTS10 gene, among others, typically confirms the diagnosis.^28^ Additionally, childhood glaucoma and ectopia lentis are often seen in homocystinuria, a genetic disease resulting in an ineffective cystathionine beta-synthase enzyme.^29^ This disease is also identifiable on genetic testing, excluding it as a possible etiology in this case.

## 4 Conclusions

We describe an unusual cause of lens abnormalities and secondary angle closure glaucoma in a child due to ciliary body cysts with no identifiable underlying genetic change. To the best of our knowledge, this represents the youngest reported case of ciliary body cysts in the literature. The management of ciliary body cysts depends largely on the associated ocular sequelae. In cases of secondary glaucoma, medical management may temporarily stabilize IOP, but ultimately more definitive surgical intervention may be required.

## Patient consent

Consent to publish the case report was not obtained. This report does not contain any personal information that could lead to the identification of the patient.
